# Changes in the Elongation Pattern of the Medial Collateral Ligament During a Single-Leg Squat After Anterior Cruciate Ligament Reconstruction

**DOI:** 10.1177/23259671241283795

**Published:** 2024-11-05

**Authors:** Piero Agostinone, Stefano Di Paolo, Gian Andrea Lucidi, Gregorio Marchiori, Marco Bontempi, Laura Bragonzoni, Vittorio Davidoni, Alberto Grassi, Stefano Zaffagnini

**Affiliations:** * 2nd Orthopaedic and Traumatologic Clinic, IRCCS Istituto Ortopedico Rizzoli, Bologna, Italy; ‡ Scienze e Tecnologie Chirurgiche, IRCCS Istituto Ortopedico Rizzoli, Bologna, Italy; § Department for Life Quality Studies, University of Bologna, Bologna, Italy; ‖ Department of Biomedical and Neuromotor Sciences, University of Bologna, Bologna, Italy; Investigation performed at IRCCS Istituto Ortopedico Rizzoli, Bologna, Italy

**Keywords:** anterior cruciate ligament, medial collateral ligament, dynamic radiographs, squat

## Abstract

**Background:**

An anterior cruciate ligament (ACL) tear impairs knee biomechanics in daily activities and potentially breaks the synergy among other knee ligaments. Previous studies have demonstrated that the biomechanics of collateral ligaments is influenced by ACL deficiency.

**Purpose:**

To investigate changes in the elongation patterns of the medial collateral ligament (MCL), lateral collateral ligament (LCL), and posterior cruciate ligament (PCL) during the execution of a single-leg squat before and after ACL reconstruction.

**Study Design:**

Descriptive laboratory study.

**Methods:**

A total of 16 patients (mean age, 24.9 ± 8.5 years) with ACL deficiency were enrolled in the study. Magnetic resonance imaging scans of the affected knees were used to produce 3-dimensional models of the tibia and femur and identify insertion sites of the MCL, LCL, and PCL. Motion capture of a single-leg squat was performed through a biplanar radiographic system. Data were acquired before ACL reconstruction and at a minimum of 18 months (mean, 22.9 ± 4.1 months) postoperatively. The centroids of the ligaments’ insertions were used to calculate the length of the investigated structures during the squat task. Absolute length (*L*), absolute length increase from the orthostatic resting position (Δ*L*), and relative length increase (Δ*L*%) were computed for each ligament, and preoperative and postoperative data were compared using the paired Student *t* test. The intraclass correlation coefficient was used to determine the reliability of the ligament insertion's identification and kinematics between 2 independent observers.

**Results:**

Significant differences were found for the MCL in absolute length increase (*P* = .047; Cohen *d* = 0.60) and relative length increase (*P* = .043; Cohen *d* = 0.61) from rest between preoperatively and postoperatively (Δ*L*_pre_ = 1.0 mm; Δ*L*_post_ = −1.1 mm; difference = 2.1 mm) at 0° to 30° of knee flexion during the descending phase of the single-leg squat. No differences were seen in the elongation patterns of the LCL or PCL from before to after ACL reconstruction.

**Conclusion:**

The MCL was significantly longer between 0° and 30° in ACL-deficient knees compared with ACL-reconstructed knees during the descending phase of a single-leg squat. No differences were identified for the LCL or PCL.

**Clinical Relevance:**

Early ACL reconstruction could have a protective effect on the MCL in combined ACL and MCL lesions.

An anterior cruciate ligament (ACL) injury is common in young, active patients.^28,33^ Trauma leading to an ACL tear is a complex event, especially in the case of noncontact mechanisms.^2,15,35^ For this reason, ACL ruptures are frequently associated with meniscal tears, cartilage lesions, and injuries to other ligaments.^15,33^ The consequences of an ACL tear have been widely investigated. It has been proven that ACL deficiency causes anterior knee laxity, increasing the risk of new knee sprains during sports activity.^2,15,33^ Moreover, a higher incidence of osteoarthritis has been recorded.^7,15,33^

Recent kinematic studies have revealed that an ACL injury influences not only anterior knee laxity but also knee internal-external rotation and tibiofemoral mediolateral alignment during the execution of active tasks and those conducted under weightbearing conditions.^1,4,10^ For these reasons, alterations in the synergic behavior of other knee ligaments could be expected in the context of ACL deficiency. In particular, a study comparing ACL-injured knees with contralateral knees demonstrated that the medial collateral ligament (MCL) length increases during knee active flexion-extension after an ACL tear; however, the lateral collateral ligament (LCL) showed significant shortening.^33^ Moreover, biomechanical changes in the posterior cruciate ligament (PCL) could be speculated, considering possible anomalies in anterior-posterior tibial alignment^6,32^ and bowing of the ligament visible in the sagittal view on magnetic resonance imaging (MRI).^13^

The aim of the present study was to investigate how ACL reconstruction influences the biomechanics of the other knee ligaments during the execution of a single-leg squat, comparing their elongation patterns before and after ACL reconstruction. We hypothesized that the MCL would be shortened, while the LCL and PCL would be more elongated after ACL reconstruction compared with preoperatively.

## Methods

This study is derived from a secondary analysis of data collected for a prospective study, whose purpose was to evaluate the clinical and biomechanical outcomes of ACL reconstruction.^10,17^ The study protocol was approved by the ethics committee of our institution, and all included patients signed informed consent forms.

The inclusion criteria for the original study were as follows: age of 16 to 50 years; complete and unilateral ACL injuries; no previous knee ligament reconstruction or repair; no concomitant PCL, posterolateral corner, LCL, or MCL lesions; and an absence of moderate or advanced knee osteoarthritis (Kellgren-Lawrence grade 3-4). For the purposes of the present study, additional inclusion criteria were single-bundle ACL reconstruction, absence of meniscal tears, availability of a complete kinematic evaluation, and quality of MRI scans sufficient to properly assess the footprints of the ligaments.

### Surgical Techniques and Rehabilitation

Patients underwent 2 different ACL reconstruction techniques with a hamstring tendon graft (gracilis and semitendinosus tendons), which in a previous study led to comparable knee kinematic patterns during the investigated motor task.^10^ All of the surgical procedures were performed by a single experienced surgeon (S.Z.), who was not aware of the purposes of the study at the time of surgery.

The first surgical technique was anatomic ACL reconstruction as described by Prodromos and Joyce.^27^ The starting point of the tibial tunnel was on the medial tibial metaphysis, inclined laterally at approximately 65° with respect to the horizontal line, directed toward the center of the native ACL tibial insertion. The harvested tendons were detached from the tibial insertion and quadrupled. A femoral half-tunnel of at least 2.5 cm was drilled from the native ACL footprint. The graft was passed through both tunnels intra-articularly and then fixed at the femur with an Endobutton (Smith+Nephew) and at the tibia with a bioabsorbable interference screw; fixation was performed at 30° of knee flexion by applying posterior drawer force. The second technique was ACL reconstruction plus lateral tenodesis as described by Marcacci et al.^25^ The semitendinosus and gracilis tendons were harvested, preserving the tibial insertion. The tibial tunnel was drilled, aiming at the posteromedial part of the ACL footprint. After a lateral incision proximal to the lateral epicondyle and dissection of the iliotibial band and intermuscular septum, the over-the-top position was identified. The graft was then passed through the tibial tunnel, into the joint, and outside of the lateral incision. Next, the graft was fixed in the over-the-top position with 2 barbed metal staples (Citieffe) with the knee at 70° of flexion by applying posterior drawer force. Finally, the distal part of the graft was passed underneath the fascial layer and fixed below the Gerdy tubercle with a metal staple.

All patients followed the same rehabilitation protocol, which did not involve the use of a knee brace. Isometric quadriceps exercises and prone hamstring stretches were allowed on the first day; passive and active flexion-extension began on the third postoperative day, initially limited to 30° of flexion and increasing 5° every day up to 90°. From the fourth week, complete range of motion was permitted. Only partial weightbearing was allowed for the first 2 weeks, then it progressively increased until patients were allowed full weightbearing in the fourth week.

Cycling, weight exercises in extension, and one-quarter squats were introduced at 4 weeks after surgery. Running on a treadmill was introduced at 2 months and aggressive strengthening and sport-specific activities after 4 months. Return to competitive sport was not allowed before the sixth month.

### Data Collection

Preoperatively and at a minimum 18-month follow-up, 1.5-T MRI (GE HealthCare) of the investigated knees was performed. At the same time, motion capture was performed using a radiographic setup for dynamic radiostereometry developed at our institute. The acquisition of images was performed in a specialized radiographic room; the specific settings were analogous to the ones described in previous studies.^1,10,16^ Overall, 2 radiography sources were placed so that the beamlines were perpendicular to each other and synchronized to acquire a pair of simultaneous radiographs (8 frames/s). Detector dimensions were 43 × 43 cm with a matrix of 1440 × 1440 pixels, and each beamline had the source-to-detector distance set to 180 cm.^5^

Within this setting, each patient was asked to perform a series of single-leg squats after the investigators carefully checked the initial position of the foot to limit the bias caused by internal-external alignment. The movement began with the patient at the center of the radiographic setting in a monopodal stance, and then, the descending phase of the squat was performed to reach the maximum degree of knee flexion allowed by the patient; subsequently, during the ascending phase, the knee was extended to reach the initial position. The dynamic radiographs were collected a single time to reduce exposure to the x-ray beams, after the patient showed confidence with the motor task, with a minimum of 2 repetitions before final acquisition.

After the motor task, radiographs of the calibration cage were acquired for data analysis, and a 3-dimensional (3D) reconstruction of the dynamic roentgen stereophotogrammetry analysis (RSA) scene was created.^1,10^ The original protocol had also included a kinematic evaluation of the uninjured contralateral knee, but for ethical reasons (x-ray exposure), additional consent of the participants was required, and most of them declined to participate in this supplementary examination.

### Data Analysis

From the preoperative MRI scans (T1-weighted sequence), 3D models of the femur and tibia were segmented using the open-source software 3D Slicer (v4.11, Slicer).^12^ The same software was also used to identify the tibial and femoral insertions of the 3 investigated ligaments: PCL, MCL, and LCL. The centroids of the ligaments’ insertions were used to calculate the length of the investigated structures during the single-leg squat, which were measured independently by 2 orthopaedic surgeons (P.A. and V.D.), who were experienced in knee ligament surgery.

**Figure 1.**
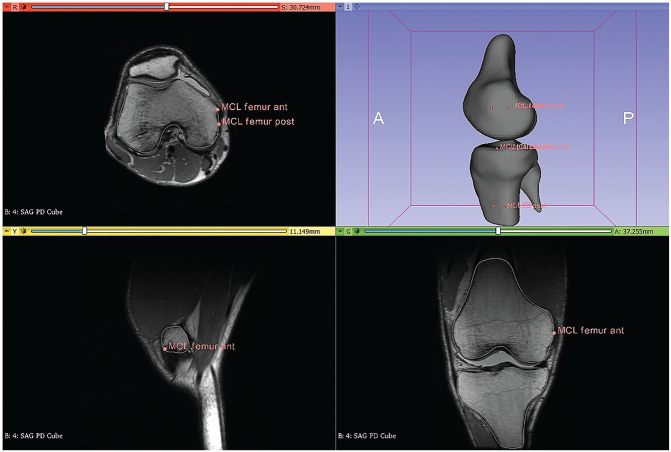
Sample images in 3D Slicer showing the identification of the MCL insertion. Selected points were used to determine the tibial and femoral centroids, and the distance between the tibial and femoral centroids was calculated as the insertion and used when measuring the length of the MCL during the single-leg squat. MCL, medial collateral ligament; 3D, 3-dimensional; ant, anterior; post, posterior.

The observers identified a series of points for each ligament that could serve as a reference to describe its insertion to calculate its length accurately. To describe the PCL, 4 reference points were identified each on the femur and tibia at the natural insertions (more anterior, more posterior, more lateral, and more medial). For the LCL, 2 points were identified on the femoral insertion and 2 on the fibular insertion (more anterior and more posterior). The same was done for the MCL, with the addition of 2 more points at the level of the tibial plateau to overcome the ligament deviation caused by the geometry of the tibia. The centroid was calculated as the mean of the coordinates of the ligament insertion points on the femur and tibia (Figure 1).^33^

**Figure 2.**
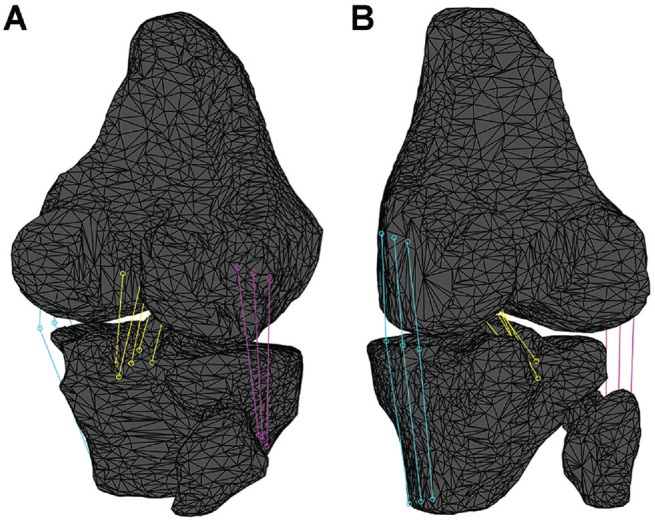
(A) Posterolateral and (B) posteromedial views of the bone segmentation and virtual reconstruction of the studied ligaments: posterior cruciate ligament (yellow), medial collateral ligament (light blue), and lateral collateral ligament (violet).

Afterward, the insertion points were projected onto the corresponding bone segments. The points’ positions relative to the patient's motion were determined using dynamic RSA and the coordinates of the landmarks. For each frame, the positions of the insertion points on the femur and tibia were calculated in the reference system of the calibration cage. The insertion points remained fixed relative to the bone segments after being projected. Points along the bundles, such as the MCL, were projected by interpolating their position relative to the insertions; as a result, the ligament smoothly deformed to follow the position of the bone segments. The length of the bundle was determined by summing the Euclidean distance between the points of the bundle from the femur to the tibia. The length of the ligament was defined as the distance between centroids (Figure 2).

Data were normalized to the peak knee flexion angle computed through the Grood and Suntay decomposition in dedicated software in Matlab (R2016a; MathWorks).^5,16,19^ Data were divided into the descending phase (from the initial standing position to peak knee flexion) and ascending phase (from peak knee flexion to the final standing position). A validated workflow with submillimetric accuracy for the model position and orientation was adopted (0.22 ± 0.46 mm and 0.26°± 0.20°, respectively).

There were 3 measures of ligament elongation calculated: absolute length (*L*), reported in millimeters; absolute length increase (Δ*L*), computed as Δ*L* = *L_i_*−*L*_rest_ in which *L_i_* is the absolute ligament length in the *i* time frame (corresponding to a specific knee flexion angle) and *L*_rest_ is the ligament length in the first frame of the acquisition, considered as the resting length of the ligament in the standing weightbearing position in full extension; and relative length increase (Δ*L*%), computed as Δ*L%* = Δ*L*/*L*_rest_ (ie, the ratio between absolute increase Δ*L* and *L*_rest_ in each time frame). The 3 measures describe different aspects of ligament elongation: *L* provides the actual length of each ligament at each knee flexion angle explored during the task, Δ*L* provides ligament stretching (Δ*L* > 0) or shortening (Δ*L* < 0) at each knee flexion angle compared to the resting position, and Δ*L*% provides ligament stretching (Δ*L*% > 0) or shortening (Δ*L*% < 0) in percentages of the resting length.

### Statistical Analysis

The intraclass correlation coefficient (ICC) was used to compute the interrater reliability of the ligament insertion's identification and elongation between the 2 independent observers. For the ligament insertions, ICC(3,1) for consistency was calculated on the centroids computed by the single points picked up by the 2 independent observers. The ICCs with 95% CIs and *P* values were computed separately for the x-axis (medial-lateral distance), y-axis (anterior-posterior distance), and z-axis (vertical distance). For elongation, ICC(3,1) for consistency was computed at specific clinically meaningful knee flexion angles: 15°, 30°, and 45°. The ICC for ligament insertion describes the reliability of the observers in identifying the reference points for analysis, while the ICC for elongation describes the reliability of the actual elongation measurements; thus, the latter accounts for the error propagation during the entire data analysis process. The interrater reliability was considered poor, fair, and excellent for ICC values <0.40, 0.40-0.75, and >0.75, respectively.^21^

Elongation data were reported as mean ± standard deviation at the different knee flexion angles. For conciseness, the results were grouped and presented for every 15° of knee flexion (eg, 0°-15°, 15°-30°, 30°-45°, etc, for the descending phase). The paired Student *t* test was used to statistically compare the preoperative and postoperative data along each frame interval of the entire motor task for each variable. Differences were considered statistically significant at *P* < .05.

An a priori power analysis was conducted to calculate an adequate sample size in G*Power (Version 3.1). The only 2 previous studies with a similar methodology and rationale were conducted on 5 and 6 knees.^26,33^ Based on their findings, a standard deviation of 6 mm was extracted for absolute length of the MCL at 30° of knee flexion.^33^ To achieve a power of 0.8 with a mean difference of 5 mm and an alpha level of .05, the minimum number of patients required was set to 14.

## Results

Overall, 16 patients (mean age, 24.9 ± 8.5 years; 14 men, 2 women) met the inclusion criteria and were included in the final analysis. The mean time from injury to imaging acquisition (RSA and MRI) was 3.4 ± 1.3 months, and the mean follow-up time was 22.9 ± 4.1 months.

**Figure A1.**
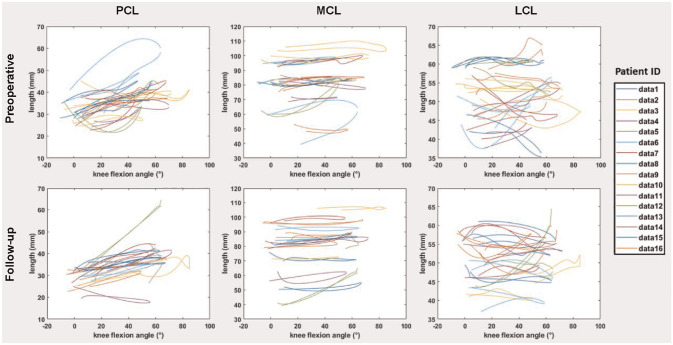
Preoperative (top) and postoperative (bottom) elongation patterns of individual patients (n = 16) during a single-leg squat at different knee flexion angles. LCL, lateral collateral ligament; MCL, medial collateral ligament; PCL, posterior cruciate ligament.

The interrater reliability for ligament insertion ranged from 0.92 to 1.00 for the PCL points, 0.23 to 1.00 for the MCL points, and 0.74 to 1.00 for the LCL points (Appendix Table A1). The interrater reliability for elongation ranged from 0.94 to 0.95 for the PCL, 0.60 to 0.76 for the MCL, and 0.61 to 0.84 for the LCL (Appendix Table A2). Elongation data for each patient are reported in Appendix Figure A1.

### Elongation Patterns of PCL and LCL

**Figure 3.**
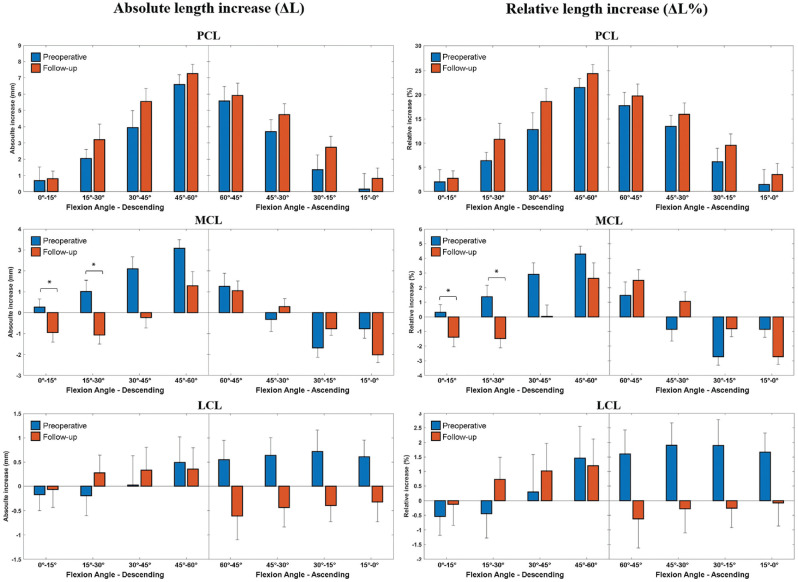
Graphical representation of the variation in knee ligament lengths during a single-leg squat before and after anterior cruciate ligament reconstruction. Error bars represent standard deviations . *A statistically significant difference was found for the medial collateral ligament between 0° and 30° of knee flexion during the descending phase (*P* < .05).

No differences in absolute length, absolute length increase, and relative length increase emerged between preoperatively and postoperatively for the PCL or LCL (Tables 1 and 2 and Figure 3).

**Table 1:** Ligament Length (in mm) During Single-Leg Squat^*a*^

	PCL				MCL				LCL			
Flexion Angle	Preoperative	Postoperative	*P*	Cohen *d*	Preoperative	Postoperative	*P*	Cohen *d*	Preoperative	Postoperative	*P*	Cohen *d*
Descending												
0°-15°	32.3 ± 0.8	30.6 ± 0.5	.255	0.33	82.6 ± 0.4	77.2 ± 0.5	.422	0.71	50.0 ± 0.3	50.7 ± 0.4	.801	0.17
15°-30°	34.2 ± 0.5	32.8 ± 1.0	.392	0.26	81.7 ± 0.6	77.5 ± 0.4	.215	0.56	51.3 ± 0.4	51.2 ± 0.4	.767	0.03
30°-45°	36.1 ± 1.1	35.5 ± 0.8	.608	0.13	82.8 ± 0.6	80.0 ± 0.5	.150	0.38	51.5 ± 0.6	51.1 ± 0.5	.698	0.01
45°-60°	38.8 ± 0.6	37.2 ± 0.6	.234	0.31	83.7 ± 0.4	81.6 ± 0.7	.236	0.31	52.0 ± 0.5	51.1 ± 0.4	.534	0.16
Ascending												
60°-45°	38.4 ± 0.9	35.8 ± 0.8	.112	0.47	81.8 ± 0.6	81.3 ± 0.5	.607	0.07	52.8 ± 0.4	50.2 ± 0.5	.146	0.54
45°-30°	35.9 ± 0.7	34.3 ± 0.7	.126	0.58	80.4 ± 0.6	78.5 ± 0.4	.971	0.38	52.1 ± 0.4	50.4 ± 0.4	.097	0.47
30°-15°	33.5 ± 0.9	32.2 ± 0.7	.285	0.54	79.0 ± 0.4	77.4 ± 0.3	.779	0.44	52.2 ± 0.4	50.4 ± 0.3	.063	0.57
15°-0°	31.4 ± 1.0	30.3 ± 0.6	.152	0.55	83.4 ± 0.5	76.0 ± 0.4	.713	2.08	52.1 ± 0.3	50.6 ± 0.4	.050	0.62

a Data are presented as mean ± SD. LCL, lateral collateral ligament; MCL, medial collateral ligament; PCL, posterior cruciate ligament.

**Table 2:** Absolute and Relative Ligament Elongation During Single-Leg Squat^*a*^

Flexion Angle	PCL				MCL				LCL			
	Preoperative	Postoperative	*P*	Cohen *d*	Preoperative	Postoperative	*P*	Cohen *d*	Preoperative	Postoperative	*P*	Cohen *d*
	Absolute Ligament Elongation, mm											
Descending												
0°-15°	0.7 ± 0.8	0.8 ± 0.5	.869	0.03	0.3 ± 0.4	−1.0 ± 0.5	**.048**	0.60	−0.2 ± 0.3	−0.1 ± 0.4	.796	0.05
15°-30°	2.1 ± 0.5	3.2 ± 1.0	.404	0.25	1.0 ± 0.6	−1.1 ± 0.4	**.043**	0.56	−0.2 ± 0.4	0.3 ± 0.4	.684	0.15
30°-45°	3.9 ± 1.1	5.5 ± 0.8	.194	0.34	2.1 ± 0.6	−0.2 ± 0.5	.081	0.47	0.0 ± 0.6	0.3 ± 0.5	.786	0.07
45°-60°	6.6 ± 0.6	7.3 ± 0.6	.616	0.13	3.1 ± 0.4	1.3 ± 0.7	.294	0.27	0.5 ± 0.5	0.3 ± 0.4	.923	0.02
Ascending												
60°-45°	5.6 ± 0.9	5.9 ± 0.8	.845	0.05	1.3 ± 0.6	1.1 ± 0.5	.868	0.02	0.6 ± 0.4	−0.6 ± 0.5	.298	0.24
45°-30°	3.7 ± 0.7	4.7 ± 0.7	.490	0.16	−0.3 ± 0.6	0.3 ± 0.4	.803	0.08	0.6 ± 0.4	−0.4 ± 0.4	.256	0.27
30°-15°	1.4 ± 0.9	2.7 ± 0.7	.356	0.21	−1.7 ± 0.4	−0.8 ± 0.3	.648	0.13	0.7 ± 0.4	−0.4 ± 0.3	.219	0.32
15°-0°	0.2 ± 1.0	0.8 ± 0.6	.398	0.13	−0.8 ± 0.5	−2.0 ± 0.4	.139	0.37	0.6 ± 0.3	−0.3 ± 0.4	.532	0.27
	Relative Ligament Elongation, %											
Descending												
0°-15°	2.0 ± 2.5	2.7 ± 1.6	.760	0.07	0.3 ± 0.5	−1.4 ± 0.7	**.043**	0.62	−0.5 ± 0.6	−0.1 ± 0.7	.680	0.10
15°-30°	6.4 ± 1.7	10.8 ± 3.3	.269	0.33	1.4 ± 0.8	−1.5 ± 0.6	**.044**	0.56	−0.4 ± 0.8	0.7 ± 0.8	.583	0.19
30°-45°	12.8 ± 3.5	18.6 ± 2.7	.143	0.39	2.9 ± 0.8	0.0 ± 0.8	.145	0.38	0.3 ± 1.3	1.0 ± 1.0	.745	0.08
45°-60°	21.5 ± 1.9	24.4 ± 1.9	.577	0.14	4.3 ± 0.6	2.6 ± 1.1	.565	0.15	1.5 ± 1.1	1.2 ± 0.9	.927	0.02
Ascending												
60°-45°	17.8 ± 2.8	19.8 ± 2.5	.759	0.09	1.5 ± 0.9	2.5 ± 0.7	.839	0.06	1.6 ± 0.8	−0.6 ± 1.0	.296	0.23
45°-30°	13.5 ± 2.3	16.0 ± 2.3	.673	0.11	−0.9 ± 0.8	1.1 ± 0.7	.621	0.13	1.9 ± 0.8	−0.3 ± 0.8	.221	0.28
30°-15°	6.1 ± 2.8	9.6 ± 2.3	.497	0.15	−2.7 ± 0.6	−0.8 ± 0.6	.529	0.17	1.9 ± 0.9	−0.3 ± 0.7	.217	0.22
15°-0°	1.5 ± 3.1	3.5 ± 2.3	.491	0.12	−0.8 ± 0.6	−2.7 ± 0.5	.093	0.49	1.7 ± 0.7	−0.1 ± 0.8	.534	0.53

a Data are presented as mean ± SD . Elongation refers to the ligament length in the resting position; positive values indicate ligament stretching, and negative values indicate ligament shortening. Boldface *P* values indicate a statistically significant difference between preoperatively and postoperatively (*P* < .05). LCL, lateral collateral ligament; MCL, medial collateral ligament; PCL, posterior cruciate ligament.

### Elongation Pattern of MCL

The MCL's absolute length increase from the rest position differed significantly preoperatively and postoperatively (*P* = .047; Cohen *d* = 0.60); preoperatively, at 0° to 30° of knee flexion, Δ*L* ranged between 0.3 and 1.0 mm (ligament stretching), and at follow-up in the same 0° to 30° flexion window, Δ*L* ranged between −1.0 and −1.1 mm (ligament shortening). The absolute difference between preoperative and postoperative values was 2.1 mm (Table 2 and Figure 3). The MCL's relative length increase from the rest position also differed significantly between preoperatively and postoperatively at 0° to 30° of flexion, ranging from 0.3% to 1.4% preoperatively (ligament stretching) to −1.4% to −1.5% at follow-up (ligament shortening) (*P* = .043; Cohen *d* = 0.61) (Table 2 and Figure 3). Overall, a 2.9% difference emerged in this phase.

## Discussion

The main finding of the present study was that the MCL was longer between 0° and 30° of flexion in ACL-deficient knees and shorter in ACL-reconstructed knees during the descending phase of a single-leg squat task. Furthermore, the LCL and PCL were not influenced by surgery.

MCL elongation was significantly greater preoperatively between 0° and 30° of knee flexion during the descending phase, with a maximum lengthening of almost 3% between 15° and 30°. These results are partially in line with those reported by Van de Velde et al,^33^ who found a lengthening of around 1.5% for the superficial MCL in ACL-deficient knees versus healthy contralateral knees in 6 patients during the execution of a quasistatic lunge. In their study, the difference was registered from 0° to 90° of knee flexion and was statistically significant for the entire range of motion. Similarly, in a previous cadaveric study,^20^ ACL transection was proven to cause a significant increase in the MCL's in situ forces, with a maximum difference registered at 30° of knee flexion. The finding should be considered relevant in view of the data reported by Victor et al^34^: The authors previously studied the elongation pattern of knee ligaments in vitro and found that the healthy superficial MCL had a maximum relative elongation of 2% between 0° and 90° of knee flexion.

The MCL could therefore be considered an agonist of the ACL from a biomechanical point of view: Thus, the MCL's role, and consequently load, become more relevant in the case of an ACL tear. This aspect was previously demonstrated by studies that evaluated anterior-posterior laxity in ACL deficiency.^17,29^ However, the knee movements are more complex than during a standard laxity test, and the influence of muscle activation and weightbearing could hide or emphasize the cornerstones of classic biomechanics. New technologies, such as dynamic radiography, video analysis, and wearable sensors, provide new insights on joint kinematics, allowing us to investigate the biomechanical implication of an injury with respect to specific motor tasks.^3,9,11^ In this regard, biplanar fluoroscopy and radiography, as a result of their high accuracy in capturing joint motion, have shown that the ACL status influences not only the anterior-posterior alignment and laxity of the knee but also the mediolateral tibial position and knee internal-external rotation.^1,8,10^ Thus, the kinematic explanation of the biomechanical anomalies of the ligaments should be seen under a broader view, not only considering the sagittal plane but all 6 degrees of freedom of the knee movement.

The present study is the first to show the role of ACL reconstruction in influencing MCL biomechanics in vivo and under weightbearing. Such findings should be considered when treating combined ACL and MCL lesions. The treatment of combined ACL and MCL tears is a controversial topic.^18,24,31,38^ Various approaches have been proposed over time, and one of the most accepted is nonoperative treatment of the MCL combined with surgery of the ACL,^30^ as not enough evidence is available to support a wider recourse of surgical treatment of the MCL or the necessity of early ACL reconstruction.^18,24,38^ The results of the present study did not justify MCL surgery or early ACL reconstruction in combined lesions, but they did suggest that ACL reconstruction could have a protective effect on MCL healing. If confirmed by further studies focused on multiligament injuries, in the future, algorithms predictive for MCL healing could permit surgeons to identify patients who would benefit from earlier ACL reconstruction in the case of concomitant ACL and MCL lesions.

In the present study, ACL reconstruction had no influence on LCL and PCL elongation. The LCL could be considered a primary stabilizer of knee varus and tibial external rotation.^22,23^ A previous in vitro study by Kanamori et al^20^ demonstrated that LCL biomechanics was influenced by ACL status. The authors evaluated the in situ forces of the collateral ligaments in ACL-intact and ACL-deficient specimens by applying a 134-N anterior tibial load and found significantly higher in situ forces in the LCL after ACL sectioning. More recently, Van de Velde et al^33^ demonstrated in vivo that ACL deficiency causes a reduction in LCL elongation during the execution of a semistatic lunge. Similarly, we expected lower stretching of the PCL before ACL reconstruction, considering that it is common to observe anterior tibial subluxation on sagittal MRI or lateral radiographs of ACL-deficient knees.^32^ The anteriorization of the tibia could be observed in both acute and chronic conditions^13^; this causes the femoral insertion to become closer to the tibial one, implying at least a reduction in the ligament length.

In the present study, there were no differences in the elongation patterns of either the LCL or PCL during the single-leg squat; however, a stabilizing effect on tibial anterior-posterior translation was found during the investigated motor task in our previous study.^10^ This was also indicated during an in vivo study by Agostinone et al^1^; moreover, except for the study by Van de Velde et al,^33^ other evidence was based on cadaveric studies or data acquired during static conditions.

### Limitations

The present study has several limitations. The first limitation was the lack of a healthy control group. However, the direct comparison between preoperative and postoperative data is a widely accepted compromise of this kind of biomechanical analysis based on radiographic movement acquisition. Moreover, no previous studies evaluating knee ligament biomechanics have simultaneously utilized an accurate motion capture tool and a large sample size in vivo and under weightbearing. Another limitation is the choice of investigating ligament behaviors by studying their length changes, which are not directly related to ligament strains. However, in vivo, it represents a valuable solution to overcome the impossibility of using invasive devices as in cadaveric settings. The examined motor task permitted the evaluation of knee biomechanics in a range of knee flexion between 0° and 60° without applying a consistent valgus load on the joint, which restricted us from investigating what happens in higher angles or in more stressful conditions. However, the collateral ligaments are thought to work heavily at low flexion angles.^20^ This factor could affect the study of PCL biomechanics, and future research based on different movements can overcome this limitation.

The collateral ligaments have previously shown different elongation patterns when divided into anterior and posterior functional bundles.^33,37^ In the present study, the method was to analyze only the central isometric region, as this is less subject to deformation. Despite the simplification, our aim was not simply to deeply distinguish the role of the bundles of the collateral ligaments but to evaluate overall ligament elongation changes in ACL deficiency. Furthermore, it is not possible to completely exclude that the differences in MCL elongation could be related to concomitant subclinical MCL tears,^36^ but all the patients involved were preoperatively examined by the same experienced orthopaedic surgeon, and neither clinical nor imaging findings suggestive of a missed preoperative MCL laxity diagnosis were identified in the follow-up evaluations.

Finally, it should be noted that, thus far, the over-the-top ACL reconstruction technique is mostly adopted in pediatric patients in the United States with the goal of respecting the physis, while its application is broader in Europe.^14^ This aspect could influence the transferability of the results to the overall patient population in the United States.

## Conclusion

In the current study, ACL reconstruction significantly reduced the MCL's length between 0° and 30° of knee flexion compared to ACL-deficient knees during the descending phase of a single-leg squat. Early ACL reconstruction could have a protective effect on the MCL in combined ACL and MCL lesions.

## Appendix

**Table A1:** Interrater Reliability for Ligament Insertions^*a*^

Reference Point	Medial-Lateral Distance		Anterior-Posterior Distance		Vertical Distance	
	ICC (95% CI)	*P*	ICC (95% CI)	*P*	ICC (95% CI)	*P*
PCL, femur	1.00 (1.00 to 1.00)	<.001	0.97 (0.93 to 0.99)	<.001	1.00 (1.00 to 1.00)	<.001
PCL, tibia	1.00 (1.00 to 1.00)	<.001	0.92 (0.80 to 0.97)	<.001	0.99 (0.96 to 0.99)	<.001
MCL, femur	0.79 (0.51 to 0.92)	<.001	0.91 (0.76 to 0.97)	<.001	0.97 (0.92 to 0.99)	<.001
MCL, tibia	0.94 (0.85 to 0.98)	<.001	0.81 (0.55 to 0.93)	<.001	0.23 (−0.27 to 0.64)	.185
MCL, joint line	1.00 (0.99 to 1.00)	<.001	0.91 (0.78 to 0.97)	<.001	0.99 (0.97 to 1.00)	<.001
LCL, femur	1.00 (1.00 to 1.00)	<.001	0.89 (0.71 to 0.96)	<.001	0.81 (0.54 to 0.93)	<.001
LCL, fibula	1.00 (1.00 to 1.00)	<.001	0.74 (0.41 to 0.90)	<.001	0.93 (0.80 to 0.97)	<.001

a Reliability was considered poor, fair, and excellent for ICC values <0.40, 0.40-0.75, and >0.75, respectively.^21^ ICC, intraclass correlation coefficient; LCL, lateral collateral ligament; MCL, medial collateral ligament; PCL, posterior cruciate ligament.

**Table A2:** Interrater Reliability for Elongation^*a*^

Ligament	15° of Flexion		30° of Flexion		45° of Flexion	
	ICC (95% CI)	*P*	ICC (95% CI)	*P*	ICC (95% CI)	*P*
PCL	0.94 (0.72 to 0.99)	<.001	0.95 (0.77 to 0.99)	<.001	0.94 (0.75 to 0.99)	<.001
MCL	0.76 (−0.06 to 0.95)	.029	0.62 (−0.52 to 0.91)	.082	0.60 (−0.61 to 0.90)	.094
LCL	0.84 (0.29 to 0.96)	.009	0.61 (−0.59 to 0.90)	.091	0.65 (−0.40 to 0.91)	.066

a Reliability was considered poor, fair, and excellent for ICC values <0.40, 0.40-0.75, and >0.75, respectively.^21^ ICC, intraclass correlation coefficient; LCL, lateral collateral ligament; MCL, medial collateral ligament; PCL, posterior cruciate ligament.
